# New insights into plasmodesmata: complex ‘protoplasmic connecting threads’

**DOI:** 10.1093/jxb/erae307

**Published:** 2024-07-13

**Authors:** Andrea A Zanini, Tessa M Burch-Smith

**Affiliations:** Donald Danforth Plant Science Center, Saint Louis, MO 63132, USA; Donald Danforth Plant Science Center, Saint Louis, MO 63132, USA; MPI of Molecular Plant Physiology, Germany

**Keywords:** Auxin, brassinosteroid, C_4_ photosynthesis, plant virus, plasmodesmata, RBOHD, systemic signaling

## Abstract

Intercellular communication in plants, as in other multicellular organisms, allows cells in tissues to coordinate their responses for development and in response to environmental stimuli. Much of this communication is facilitated by plasmodesmata (PD), consisting of membranes and cytoplasm, that connect adjacent cells to each other. PD have long been viewed as passive conduits for the movement of a variety of metabolites and molecular cargoes, but this perception has been changing over the last two decades or so. Research from the last few years has revealed the importance of PD as signaling hubs and as crucial players in hormone signaling. The adoption of advanced biochemical approaches, molecular tools, and high-resolution imaging modalities has led to several recent breakthroughs in our understanding of the roles of PD, revealing the structural and regulatory complexity of these ‘protoplasmic connecting threads’. We highlight several of these findings that we think well illustrate the current understanding of PD as functioning at the nexus of plant physiology, development, and acclimation to the environment.

## Teaser

Leveraging the latest advances in modern molecular biology, proteomics, and cutting-edge imaging technologies, researchers are delving into the intricate details of plasmodesmata form, formation, and function, revealing essential functions.

## Introduction

Plasmodesmata (PD) are intercellular nanopores that traverse the cell wall of plant cells, connecting adjacent cells and providing a route for movement of molecules between cells. Although PD were first described >100 years ago (see discussion in Gunning, 1976), they are still viewed as ‘protoplasmic connecting threads’, and we still have a limited understanding of their biogenesis, composition/ultrastructure, how cargo move through these structures, and even their functions. However, with the increasing use of tools of modern molecular biology, proteomics, and especially advanced imaging modalities, we are finally probing critical aspects of the form, formation, and function of PD. Over the last few decades, the community studying PD has provided experimental evidence for PD as conduits for the trafficking of various molecules, including ions, hormones, and small RNA molecules, enabling direct communication and coordination between plant cells. Thus, they play a crucial role in various physiological processes, such as nutrient transport, developmental signaling, and defense responses. Recent findings have reinforced the concept that they are essential for the integrity and function of plant tissues and contribute significantly to the adaptability and resilience of plants in their environment.

PD comprise a continuous plasma membrane (PM) that extends through the cell wall, connecting neighboring cells. A common constituent of PD in land plants is a strand of the endoplasmic reticulum (ER), called the desmotubule, that spans the center of the plasmodesmatal opening (Box 1). The space between the PM and the desmotubule is known as the cytoplasmic sleeve, and to date it has been identified as the main route for intercellular trafficking via PD. The last decade or so has yielded valuable insights into the PM lipid and protein constituents of PD, such that PD are now viewed as highly specialized membrane contact sites (MCS; Brault *et al.*, 2019; Petit *et al*., 2019; Ishikawa *et al.*, 2020) (Box 1). In this short article, we will highlight some of the progress in elucidating important aspects of the composition and function of PD made in the last few years that extend their roles into signaling and hormone distribution (Box 2). These findings highlight the complexity of PD and uncover even more questions about how these structures are regulated and contribute to integrated tissue- and organism-level responses.

Box 1. **Structure and composition of plasmodesmata**

**Figure 1.**
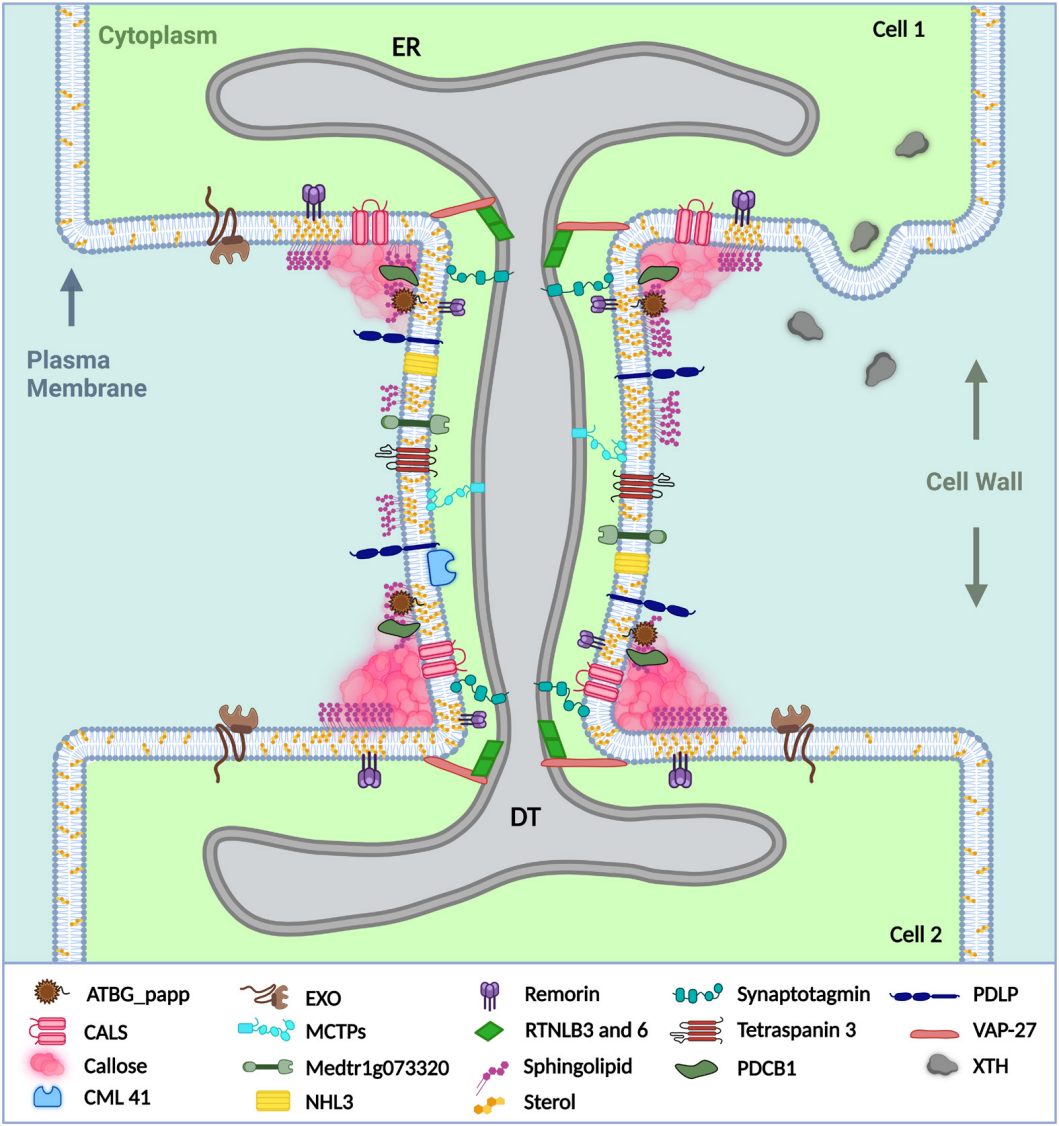
Schematic representation of two neighboring plant cells connected through a plasmodesma The structure and composition of a plasmodesma is outlined in detail according to the recent proteomic studies discussed in this review. Note that the illustration is not drawn to scale. Created with BioRender.

PD are nanopores with outer diameters ranging from 25 nm to 50 nm depending on the tissue and species, and they extend for the thickness of the cell wall, typically ~100 nm. The center of PD of land plants is usually occupied by an appressed strand of cortical ER called the desmotubule (DT) whose lumen is largely occluded by proteins (Tilney *et al.*, 1991) (Fig. 1). The cytosol-filled space between the DT and PM is called the cytoplasmic sleeve or annulus, and it is further divided into nanochannels by the spoke proteins. A nanochannel is typically only 2–3 nm wide, approximately the soluble radius of GFP, but the cytoplasmic sleeve is likely to be the main route for plasmodesmatal trafficking.

Over 1300 proteins were identified in the putative plasmodesmatal proteome of *Arabidopsis thaliana* suspension cell cultures, and localization of several to PD has been confirmed (Fernandez-Calvino *et al.*, 2011). The plasmodesmatal association of the confirmed PD-localized proteins is supported by recent studies from other plants including moss (Leijon *et al.*, 2018; Gombos *et al.*, 2023; Johnston *et al.*, 2023). Protein functions most often associated with plasmodesmatal proteins include callose metabolism [callose synthases (CALS), β-1,3-glucanases, callose binding], stress responses (peroxidases, calreticulin, LRR kinases), and membrane trafficking (syntaxins, VAMPs, and SNAP proteins). Multiple C2 domains and transmembrane region proteins (Brault *et al.*, 2019) are constituents of PD and they have been designated as the likely spokes of PD (Ding *et al.*, 1992; Brault *et al.*, 2019). The spokes of PD probably control the spacing between the DT and PM, and this distance is correlated with developmental states of the PD (Nicolas *et al.*, 2017). Callose synthases associate with signaling nodes formed by PDLPs and NHL3 (Tee *et al.*, 2023). CML41 signals through PDLPs during defense responses, leading to callose deposition at PD (Xu *et al.*, 2017). Numerous cell wall-associated proteins also localize to PD. EXO and EXO-like proteins and members of the cell wall-modifying XTH family are also found in moss PD (Gombos *et al.*, 2023). The kinase Medtr1g073320 is part of the *M. truncatula* plasmodesmatal proteome (Kirk *et al.*, 2022).

The lipid composition of plasmodesmatal PMs is distinct from that of the bulk PM. The PMs of PD from Arabidopsis suspension cells are enriched in sterols and sphingolipids with saturated very long chain fatty acids (Grison *et al.*, 2015). This is supported by the presence of the lipid microdomain marker protein remorin (Raffaele *et al.*, 2009; Simon-Plas *et al.*, 2011), and numerous GPI-anchored proteins in the plasmodesmatal proteome (Fernandez-Calvino *et al.*, 2011). Indeed, several ER–PM contact site proteins were identified as part of plasmodesmatal proteomes including synaptotagmins and vesicle-associated membrane protein-associated proteins (VAPs) (Schapire *et al.*, 2008; Wang *et al.*, 2014).

## Composition of plasmodesmata

The protein and lipid composition of PD is still being elucidated, although much progress has been made in the last several years (Box 1). Consistent with a single evolutionary origin of PD in land plants (Brunkard and Zambryski. 2017), recent analyses suggest that the plasmodesmatal protein composition is largely conserved across evolution. Analysis of plasmodesmatal proteomes from the moss *Physcomitrium patens* revealed commonalities with known proteomes from existing angiosperm plasmodesmatal proteomes. Johnston *et al.* (2023) generated an Arabidopsis proteome from mature leaves and another from mixed tissues of the moss *P. patens*. The localization of candidate proteins in PD was confirmed by confocal microscopy. A comparative analysis using previous proteomes from Arabidopsis (Fernandez-Calvino *et al.*, 2011; Brault *et al.*, 2019) and *Populus trichocarpa* (poplar) (Leijon *et al.*, 2018) analysis generated 16 orthogroups of plasmodesmal proteins conserved among these organisms. Several of these orthogroups contained proteins with verified plasmodesmatal localization, for example a glycosylphosphatidylinositol (GPI)-anchored β-1,3-glucanase and a multiple C2 domains and transmembrane region protein (MCTP) (Box 1).

Taking their attempt to identify bona fide plasmodesmatal proteins further, Gombos *et al.* (2023) leveraged an iterative bioinformatic and experimental approach based on previously published plasmodesmatal proteomes and included experimental verification of localization of identified proteins to PD. A resource that describes the analytical pipeline used for this work is publicly available (https://pddb.uni-hohenheim.de/). Using this approach, they identified members of the GH17 family as moss plasmodesmatal proteins, a finding supported by the identification of callose at moss PD (Renzaglia *et al.*, 2024). Other cell wall-modifying enzymes including XYLOGLUCAN ENDOTRANSGLYCOSYLASE/HYDROLASE (XTH) proteins were also found as part of the moss proteome. An interesting group of moss plasmodesma-localized proteins (PDLPs) is the EXORDIUM (EXO) and EXORDIUM-LIKE proteins. These proteins have been implicated in brassinosteroid (BR) signaling and cell expansion (Coll-Garcia *et al.*, 2004; Schroder *et al.*, 2012), an important consideration we revisit later in this discussion.

Another bioinformatics tool, PIP1, makes publicly available an *in silico* pipeline to identify plasmodesmatal proteins based on homology (https://github.com/PhilPlantMan/PIP1/) (Kirk *et al.*, 2022). The potential utility of this pipeline was demonstrated by the verification of localization of Medtr1g073320 in PD identified from the *Medicago truncatula* genome. PIP1 also leverages interaction networks to identify experimental conditions that could potentially regulate PD. The advent of these tools and approaches represents an important development in the field of PD and should increase the frequency with which potential plasmodesmatal proteins from diverse plant species are identified.

Alongside questions about the composition of PD are those regarding how cellular cargo is transported to and through PD. Plant viruses have long been used as probes to answer these questions (Heinlein, 2015; Reagan and Burch-Smith, 2020). Identification of plasmodesmal targeting signals on the PLASMODESMATA LOCATED PROTEIN5 (PDLP5) protein and several plasmodesmatal-localized receptor kinases was accomplished by novel computational approaches. This non-conventional signal consists of amino acid sequences in the extracellular region that is located close to the membrane, and these signals could target PM-localized receptor kinases to PD (Luna *et al.*, 2023). This marks a breakthrough in understanding how the plasmodesmatal proteome is assembled, extending work identifying the GPI anchor as a likely plasmodesma-targeting sequence on plant proteins (Zavaliev *et al.*, 2016) and the identification of plasmodesma-targeting sequences of viral movement proteins (Yuan *et al.*, 2016, 2018). Interestingly the DUF26 domains of the PDLPs are structurally similar to carbohydrate-binding lectins, perhaps explaining their involvement in callose homeostasis (Vaattovaara *et al.*, 2019). How mobile cellular RNA cargo is transported in cells to arrive at PD for intercellular and then systemic trafficking has also been examined. Using FLOWERING LOCUS T (FT) mRNA as a probe, cyclophilins of the ROC family were identified as potential adaptors that connect RNA molecules to vesicles that traffic along the cytoskeleton for delivery to PD (Luo *et al.*, 2024). The use of these molecular tools in combination with new computational approaches promises further breakthroughs in unraveling the mechanisms of plasmodesmatal trafficking.

## Environmental regulation of plasmodesmata

Possibly because of their location within the cell wall, PD are often viewed as static organelles, rather like plumbing in a concrete structure. Instead, PD, both as a collective group and as individuals, are dynamic and respond to developmental and environmental signals. This has been well documented by studies of PD in the cambium of *Populus nigra* over seasons (Fuchs *et al.*, 2010). These studies and others have shown that the numbers of PD at a given cell–cell interface can change in response to environmental and developmental signals, presumably to provide the optimal degree of connectivity for tissue function.

This concept was reinforced by the exciting demonstration that in C_4_ plants the number of PD connecting the mesophyll (M) and bundle sheath (BS) changes depending on the light conditions (Schreier *et al.*, 2024). The increased plasmodesmal connectivity is a conserved trait among C_4_ plants, resulting in 8- to 13-fold higher plasmodesmatal frequency in C_4_ M–BS interfaces compared with closely related C_3_ species. Notably, inhibiting photochemical reactions and chloroplast development significantly reduced formation of PD during de-etiolation, yet this effect is mitigated by the exogenous supply of sucrose. The necessity of functional chloroplasts becomes evident as sucrose alone proved insufficient to support formation of PD in plants lacking functional chloroplasts (Schreier *et al.*, 2024). These observations extend previous knowledge on the relationships between light and plasmodesmatal development, and between chloroplasts and PD in leaves of C_4_ plants (Wang *et al.*, 2017; Danila *et al.*, 2019). It is important to note that the observations about increases in plasmodesmatal density made by Schreier *et al.* (2024) required the application of high-resolution, three-dimensional imaging (volume EM). This is exemplary of the utility of such techniques and the necessity for the wider, skilled adoption of such approaches by those who study PD. However, caution must be exercised that these tools, which can be expensive in terms of instrument costs and user time, do not make other valid approaches obsolete.

Temperature is another environmental factor that influences PD. This has been well documented to be the case for responses to chilling, where dynamic regulation of callose levels at PD have been shown to modulate intercellular flux in meristems to control dormancy responses (Rinne and van der Schoot, 1998; Bilska and Sowinski, 2010; Rinne *et al.*, 2018; Tylewicz *et al.*, 2018; Gao *et al.*, 2021). While induction of callose at PD and dormancy involve abscisic acid (ABA), release from dormancy involves gibberellic acid (Gao *et al.*, 2021). Recent studies in Arabidopsis reveal that elevated temperatures (30 °C) induce callose accumulation at PD at the sieve element–phloem-pole pericycle (PPP) and PPP–endodermal interfaces where sugars are exiting the phloem (unloading; Liu *et al.*, 2022). This likely defect in sugar export due to reduced plasmodesma-mediated intercellular trafficking in the growing parts of the root was proposed to be the cause of slower root growth in plants treated with higher temperatures. Plasmodesma-mediated trafficking in angiosperm wood, in the xylem parenchyma, is dependent on the season, indicating responses to environmental signaling (Slupianek *et al.*, 2023).

The dynamics of regulation of PD in growth transition from slow to fast growth after dormancy release were recently studied, revealing a novel mechanism for regulating the callose level at PD. *Lillium* spp. (lily) VERNALIZATION INSENSITIVE 3-LIKE 1 (LoVIL1), part of the Polycomb Repressive Complex that methylates target loci, promotes plasmodesmatal permeability via epigenetic repression of *CALLOSE SYNTHASE 3* (*LoCALS3*) expression (Pan *et al.*, 2023). This enables cellular communication through PD and sugar symplastic transport in shoot apical meristems (SAMs), activating cell activity and facilitating bulb growth transition. In addition, a novel transcription factor, LoNFYA7, may also play a role in H3K9ac regulation through an unknown mechanism, with H3K9ac potentially fine-tuning LoNFYA7 transcription. It will be interesting to determine whether such a strategy to regulate callose levels at PD is more widespread, potentially opening up new avenues for manipulating PD for biotechnology.

## Function of plasmodesmata

### Development

Auxin is well known for its critical roles in plant development, and the PIN, AUX1/LAX, and ABCB proteins are crucial for the active transmembrane transport of auxin (Zhang *et al.*, 2023). However, cell-to-cell trafficking of auxin via PD is also essential for establishing auxin gradients (Gao *et al.*, 2020; Mellor *et al.*, 2020; Band, 2021). These findings have explained previous observations about the involvement of auxin and PD in root development (Han *et al.*, 2014). They have also been supported by numerous subsequent publications that agree with the findings that the PD–auxin axis is crucial for plant development. Lateral root development (Sager *et al.*, 2020), cell division of the root apical meristem (Olatunji *et al.*, 2023), and root branching in response to transient water stress (xerobranching) (Mehra *et al.*, 2022) have all recently been shown to require PD-mediated auxin transport. It should be noted that plasmodesmatal transport of ABA is critical for xerobranching. Importantly, ABA could also negatively regulate PD-mediated intercellular trafficking by inducing expression of *PDLP2* and *3*, leading to callose accumulation at PD (Mehra *et al.*, 2022). This, in turn, limited auxin cell-to-cell transport via PD and altered radial auxin gradients and root growth.

The role of PD and intercellular trafficking of auxin in leaf development has recently been investigated. Using the *gnome* (*gn*) mutants as a starting point, the roles of auxin signaling and transport in leaf vein patterning were investigated (Linh and Scarpella, 2022). The inclusion of mutants with defects in plasmodesmatal callose metabolism (*gsl8*, *cals3*) in the analysis demonstrated that the movement of auxin or auxin-generated signals via PD in addition to polar auxin transport was critical for leaf vein patterning. Leaf hyponastic responses have long been known to be auxin dependent. Auxin trafficking via PD was found to act in parallel to and non-redundantly with polar auxin transport by the PIN proteins (Li *et al.*, 2023).

Another exciting recent development concerns the relationship between PD and BR hormones (Wang *et al.*, 2023). Studies in Arabidopsis revealed that BRs can move between via PD. More specifically, the movement of BR precursors over short distances between root cells via PD is necessary for the completion of BR biosynthesis. Further, plasmodesma permeability was regulated by BR levels, with higher BR levels leading to decreased permeability. As was observed for other hormones, callose levels at PD were the determining factor in establishing plasmodesmatal permeability to BR factors and, although the endogenous players in BR-mediated regulation of PD are yet to be determined, BR was found to transcriptionally regulate the expression of many callose metabolism genes. Given the well-established interplay between auxin and BR in plant cell division, it will be very interesting to examine their cooperative effects on PD, if any.

### Systemic signaling

PD have long been understood to have roles in systemic transport of signals (see discussions in Gunning, 1976). It has been proposed that reactive oxygen species (ROS) can travel cell-to-cell via PD and act as systemic signals in response to stresses including high light (Fichman *et al.*, 2021). Apoplastic ROS generated by respiratory burst oxidase homolog D (RBOHD) was found to increase plasmodesmatal permeability through its effects on PDLPs to allow wave propagation. The finding that *pdlp1* and *pdlp5* mutants have lost or have severely reduced propagation of systemic signaling is somewhat surprising since these mutants had no change in intercellular trafficking (Thomas *et al.*, 2008) or had increased intercellular trafficking compared with wild-type plants (Lee *et al.*, 2011). It therefore is unclear how the systemic signals are propagated since these findings would suggest that ‘closed PD’ with greatly reduced cytoplasmic space between the desmotubule and PM (Box 1) are the conduits for the systemic signal. Are the plasmodesmatal membranes the route for signal transmission? Further complicating the issue, is that ROS have been identified as a potent inducer of callose accumulation at PD (Tee *et al.*, 2023; Vu *et al.*, 2023, and references therein), again leading to ‘closed PD’ and reduced intercellular trafficking. This raises a question for careful examination and consideration: do the ROS produced in response to different stresses have distinct effects on PD, opening or closing PD to regulate systemic signaling?

Several Ca^2+^-permeable channels were also found to be critical for amplification or maintenance of the ROS signal. The effect of the ROS wave is to initiate acclimation responses that allow plants to cope with the stress that instigated the ROS wave. A mutant screen has identified H_2_O_2_-INDUCED CA^2+^ INCREASES 1 (HPCA1) as the ROS receptor in Arabidopsis plants (Fichman *et al.*, 2022). HPCA1 is part of a signaling circuit induced by high light stress or bacterial infection that includes Ca^2+^ channels and sensors that regulate ROS production by RBOHD, coordinating systemic ROS and Ca^2+^ signaling (Box 2). These and other studies (e.g. Toyota *et al.*, 2018) suggest that symplastic pathways via PD are critical for systemic Ca^2+^ signaling. However, recent evidence suggests that wound-induced Ca^2+^ waves are instead propagated systemically via the apoplast driven by bulk flow and dependent on amino acid diffusion (Bellandi *et al.*, 2022). Importantly, Bellandi and co-workers found that PD were not involved in Ca^2+^ wave propagation since accumulation of callose at PD, rendering them ‘closed’, had no effect on systemic wound signaling. Further, the wound-induced Ca^2+^ wave did not involve RBOHD/F, suggesting a distinct means for signal propagation from that described for high light- or biotic stress-induced waves. Together, these contrasting findings reveal the complexity of systemic signaling, and highlight that both the symplastic and apoplastic routes may have specific contributions to signaling.

Box 2. **Key findings related to plasmodesmata function beyond ‘protoplasmic connecting threads’**

**Figure 2.**
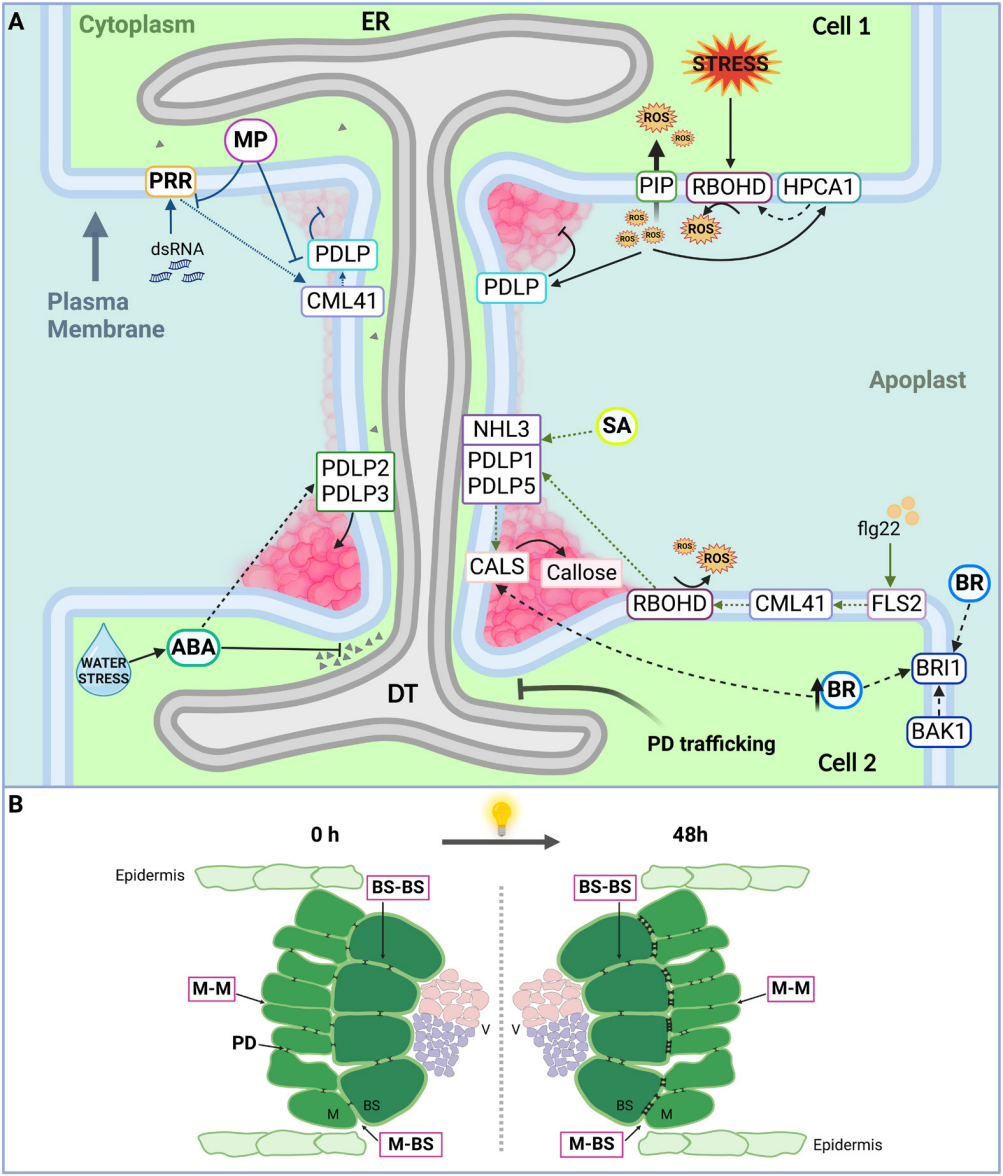
Schematic representation of recent key findings on the regulation of plasmodesmata. (A) Recently reported actors in regulating plasmodesmatal opening and closing in response tobiotic and abiotic stress. (B) Photosynthetic tissues in a C4 plant in cross-section showing the light-induced increase in the PD frequency at the M-BS interface. Note that the illustration is not drawn to scale. Created with BioRender.

- Tee et al. (2023) identified a critical node in immune responses, the PDLP–NHL3 complex, which serves as a central integrator, orchestrating multiple signaling cascades to effectively regulate closure of PD by callose synthesis (A).
- Wang *et al*. (2023) discovered a previously unknown transport mode for steroidal hormones, where the intercellular BR content negatively regulates plasmodesmatal permeability via callose deposition, affecting its mobility and biosynthesis (A).
- Mehra *et al*. (2022) found that ABA moves from cell to cell via PD and regulates callose levels to control auxin flux in response to water stress in root xerobranching (A, auxin represented by triangles).
- Fichman *et al*. (2022) reveal that HPCA1, a leucine-rich-repeat receptor-like kinase in Arabidopsis, plays a key role in sensing and coordinating systemic cell-to-cell ROS and calcium signals required for plant acclimation to stress (A).
- Huang *et al*. (2023) uncovered that dsRNA activates a signaling mechanism involving CML41 and PDLPs in PD to control their permeability via callose accumulation during PTI. Viral MPs can inhibit dsRNA-mediated changes in PD (A).
- Schreier *et al.* (2024) demonstrated an enhanced formation of PD between M and BS cells in C_4_ plants, which is coordinated by light and dependent on photosynthesis. The increased plasmodesmatal connectivity is likely to be a conserved trait found in both C_4_ plants, dicotyledons and monocotyledons (B) (Fig. 2).

Another recent study brings the interplay between symplastic and apoplastic signaling into focus. Using Arabidopsis *radical induced cell death1* (*rcd1*) as a system to study root-to-shoot signaling, Jin and co-workers built on their previous work (Liang *et al.*, 2012, 2014; Jin *et al.*, 2022) to examine how extracellular ROS, specifically superoxide, act to control the symplastic trafficking of small RNAs. Their findings suggest that extracellular superoxide impacts phytosterol levels in the plasma membrane. These changes then lead to altered numbers of PD and a concomitant change in symplastic trafficking, coupled with defects in the development of pits that regulate sap transport.

### Defense

Biotic and abiotic stresses trigger a cascade of defense reactions and modulation of the intercellular transport in plant tissues via changes in plasmodesmatal permeability. During plant–virus interaction, viral movement proteins (MPs) ensure the transport of virions or viral nucleic acid–protein complexes using independently evolved strategies. These strategies and the host proteins co-opted for viral intercellular spread have been intensely investigated (Heinlein, 2015; Reagan and Burch-Smith, 2020). More recently, ER- and desmotubule-associated reticulons (Box 1), RTN3 and 6, were found to directly interact with viral MPs from cucumber mosaic virus (CMV), and several other viruses (Tilsner and Kriechbaumer, 2022). However, interaction between CMV MP and RTN3 reduced the ability of the MP to induce increased intercellular movement of the green fluorescent protein (GFP) probe, suggesting that the interaction modulates MP function. Several other ER proteins also interact with the CMV MP (Ham *et al.*, 2023). The interaction between the MP and the ER luminal binding proteins (BiPs) was bridged by the integral membrane AtERdj2a. In contrast to the role of reticulons in limiting MP-mediated increase in intercellular trafficking, BiPs were necessary for viral systemic spread, presumably via their role in cell-to-cell viral spread. The ER lumen localization of BiPs reported in this study challenges the long-held notion that the ER lumen does not occupy the desmotubule, which is instead occluded by proteins that make possible the extreme membrane curvature of the structure. Nonetheless, it is consistent with a few demonstrations of ER lumen continuity between cells (Tilsner *et al.*, 2011). This highlights another aspect of plasmodesma structure that requires further investigation.

Retrograde signaling from chloroplast to nucleus has previously been proposed to control plasmodesma formation and regulation (Burch-Smith *et al.*, 2011; Burch-Smith and Zambryski, 2012). Supporting this idea, potato virus X (PVX) infection triggered Kunitz peptidase inhibitor-like protein (KPILP) expression, which repressed expression of nuclear-encoded chloroplast genes associated with retrograde signaling (Ershova *et al.*, 2022). This was also associated with reduced photoassimilate accumulation and increased intercellular trafficking. Over the last decade or so, numerous studies have highlighted the chloroplast as a critical player in plant–virus interactions (Delprato *et al.*, 2015; Bhattacharyya and Chakraborty, 2018; Bwalya and Kim, 2023). However, it remains to be established if chloroplast-to-nucleus retrograde signaling is commonly involved.

As with revealing the effects of light on development of PD, 3D electron microscopy has also proven vital in revealing more about how PD determine the outcome of plant–virus interactions. Using the phloem-limited reovirus, Southern rice black-streaked dwarf virus (SRBSDV), Lv *et al.* (2024) employed serial block face-SEM to illuminate the role of PD and irregular PD in preventing viral spread out of the vascularized tumors that are the sites of viral replication. PD with drastic modifications to their typical structures were also observed in those samples (although the authors chose to designate those as ‘flexible gateways’). This study exemplifies a promising way to better understand PD in defense, as plasmodesmatal density and viral infection in large numbers of cells can be simultaneously measured, and demonstrates the high resolution at which plasmodesma structure can be determined. Studies of PD-associated bodies (PAMBs) formed by the Hibiscus green spot virus (HGSV) MPs are another example of the power of volume (3D) EM to elucidate the relationship between PD and viruses (Atabekova *et al.*, 2023).

dsRNA is well known for its role in inducing the antiviral RNA silencing machinery (Niehl and Heinlein 2019; Ding, 2023). Building on the finding that dsRNA can also induce other types of defense, namely pattern-triggered immunity (PTI) (Korner *et al*., 2013; Niehl *et al*., 2016), Huang *et al.* (2023) characterized the cellular machinery involved in dsRNA-triggered PTI. Callose accumulation at PD and calcium signaling via CALMODULIN-LIKE protein 41 (CML41) (Xu *et al.*, 2017) were identified as critical components of the PTI response, while ROS, specifically H_2_O_2_, and mitogen-activated protein kinases (MAPKs) 3 and 6 were not implicated, in contrast to most other PTI responses (Lang and Colcombet, 2020). Further, PDLP1, 2, and 3 were identified as involved in callose accumulation but not PDLP5 (Box 2).

PD also have pivotal roles in responding to pathogens besides viruses. Closure of PD in response to microbial elicitors such as chitin and flg22 is orchestrated by specific receptor complexes and modulators; however, notably both chitin and flg22 responses converge on callose synthesis with an apparent central node of response. Recent investigations revealed that one such node involves the interaction of PDLPs and NON-RACE SPECIFIC DISEASE RESISTANCE/HIN1 HAIRPIN-INDUCED-LIKE protein 3 (NHL3), upstream of CALLOSE SYNTHASE 1 (CALS1) activation (Tee *et al.*, 2023) (Box 2). Salicylic aid (SA) elicits a signaling cascade that converges on microbial-triggered responses downstream of ROS production by RBOHD, an NADPH oxidase. PDLP1 and PDLP5, SA-triggered responses, interact with NHL3, establishing a central integrator for plasmodesmal immune signaling (Tee *et al.*, 2023). This suggests that NHL3 interacts with PDLPs to transmit information and activate callose synthesis, ultimately leading to closure of PD in response to diverse immune elicitors.

Effector proteins from fungal pathogens have recently been found to traffic between cells via PD (Cao *et al.*, 2018; Blekemolen *et al.*, 2022; Talbi *et al.*, 2023; Ohtsu *et al.*, 2024), as have those from bacterial pathogens (Aung *et al.*, 2020; Li *et al.*, 2021). Other effectors have been shown to localize to PD and interfere with callose deposition concomitant with increased intercellular trafficking (Tomczynska *et al.*, 2020; Rahman *et al.*, 2021). Together with the myriad studies on virus–PD interactions, these findings reinforce the idea that PD have important roles in the establishment of pathogen infections of plant hosts. In the future we expect that the roles of PD in beneficial host–microbe interactions will be investigated with similar intensity to that which has been given to understanding pathogenic interactions. While several studies have revealed the important roles of PD and symplastic trafficking in nodule formation and function, molecular details are lacking. Happily, progress is being made in this area. A member of the TETRASPANIN family of proteins known to associate with PD, TETRASPANIN8-1, was necessary for nodule formation by *Rhizobium tropici* and arbuscule formation by *Rhizophagus irregularis* on common bean (*Phaseolus vulgaris*) (Parra-Aguilar *et al.*, 2023). While TETRASPANIN8-1 does not itself localize to PD, this finding raises the possibility that PD-associated TETRASPANINs may have similar roles in plant–microbe interactions.

## Conclusion

With the advent and adoption of new analytical techniques, we expect the next few years to continue to reveal much more about the form, formation, and function of PD. As the finding that PD are critical for correct establishment of auxin gradients that are necessary for development has revealed much about the role of PD, we expect that further investigation of the interplay between PD and other phytohormones will also illuminate other critical aspects of the contributions of PD to plant survival. The rise of advanced microscopy techniques will provide unprecedented insights into the 3D architecture and dynamics of PD. By unraveling the intricate composition of PD with precision, new clues will emerge to elucidate a wide range of mechanisms underlying plant growth, adaptation, and interaction with pathogens, thus advancing agricultural practices and biotechnological applications.

## References

[CIT0001] Atabekova AK , Golyshev SA, Lezzhov AA, et al. 2023. Fine structure of plasmodesmata-associated membrane bodies formed by viral movement protein. Plants 12, 4100. 38140427 10.3390/plants12244100 PMC10747570

[CIT0002] Aung K , Kim P, Li Z, Joe A, Kvitko B, Alfano JR, He SY. 2020. Pathogenic bacteria target plant plasmodesmata to colonize and invade surrounding tissues. The Plant Cell 32, 595–611. 31888968 10.1105/tpc.19.00707 PMC7054039

[CIT0003] Band LR. 2021. Auxin fluxes through plasmodesmata. New Phytologist 231, 1686–1692. 34053083 10.1111/nph.17517

[CIT0004] Bellandi A , Papp D, Breakspear A, et al. 2022. Diffusion and bulk flow of amino acids mediate calcium waves in plants. Science Advances 8, eabo6693. 36269836 10.1126/sciadv.abo6693 PMC9586480

[CIT0005] Bhattacharyya D , Chakraborty S. 2018. Chloroplast: the Trojan horse in plant–virus interaction. Molecular Plant Pathology 19, 504–518. 28056496 10.1111/mpp.12533 PMC6638057

[CIT0006] Bilska A , Sowinski P. 2010. Closure of plasmodesmata in maize (*Zea mays*) at low temperature: a new mechanism for inhibition of photosynthesis. Annals of Botany 106, 675–686. 20880933 10.1093/aob/mcq169 PMC2958785

[CIT0007] Blekemolen MC , Cao L, Tintor N, de Groot T, Papp D, Faulkner C, Takken FLW. 2022. The primary function of Six5 of *Fusarium oxysporum* is to facilitate Avr2 activity by together manipulating the size exclusion limit of plasmodesmata. Frontiers in Plant Science 13, 910594. 35968143 10.3389/fpls.2022.910594 PMC9373983

[CIT0008] Brault ML , Petit JD, Immel F, et al. 2019. Multiple C2 domains and transmembrane region proteins (MCTPs) tether membranes at plasmodesmata. EMBO Reports 20, e47182. 31286648 10.15252/embr.201847182 PMC6680132

[CIT0009] Brunkard JO , Zambryski PC. 2017. Plasmodesmata enable multicellularity: new insights into their evolution, biogenesis, and functions in development and immunity. Current Opinion in Plant Biology 35, 76–83. 27889635 10.1016/j.pbi.2016.11.007

[CIT0010] Burch-Smith TM , Brunkard JO, Choi YG, Zambryski PC. 2011. Organelle–nucleus cross-talk regulates plant intercellular communication via plasmodesmata.. Proceedings of the National Academy of Sciences, USA 108, E1451–E1460. 10.1073/pnas.1117226108 PMC3251100 22106293

[CIT0011] Burch-Smith TM , Zambryski PC. 2012. Plasmodesmata paradigm shift: regulation from without versus within. Annual Review of Plant Biology 63, 239–260. 10.1146/annurev-arplant-042811-105453 22136566

[CIT0012] Bwalya J , Kim KH. 2023. The crucial role of chloroplast-related proteins in viral genome replication and host defense against positive-sense single-stranded RNA viruses. Plant Pathology Journal 39, 28–38. 36760047 10.5423/PPJ.RW.10.2022.0139 PMC9929168

[CIT0013] Cao L , Blekemolen MC, Tintor N, Cornelissen BJC, Takken FLW. 2018. The *Fusarium oxysporum* Avr2–Six5 effector pair alters plasmodesmatal exclusion selectivity to facilitate cell-to-cell movement of Avr2. Molecular Plant 11, 691–705. 29481865 10.1016/j.molp.2018.02.011

[CIT0014] Coll-Garcia D , Mazuch J, Altmann T, Mussig C. 2004. EXORDIUM regulates brassinosteroid-responsive genes. FEBS Letters 563, 82–86. 15063727 10.1016/S0014-5793(04)00255-8

[CIT0015] Danila FR , Quick WP, White RG, von Caemmerer S, Furbank RT. 2019. Response of plasmodesmata formation in leaves of C_4_ grasses to growth irradiance. Plant, Cell & Environment 42, 2482–2494. 10.1111/pce.13558 30965390

[CIT0016] Delprato ML , Krapp AR, Carrillo N. 2015. Green light to plant responses to pathogens: the role of chloroplast light-dependent signaling in biotic stress. Photochemistry and Photobiology 91, 1004–1011. 25989185 10.1111/php.12466

[CIT0017] Ding B , Turgeon R, Parthasarathy MV. 1992. Substructure of freeze-substituted plasmodesmata. Protoplasma 169, 28–41.

[CIT0018] Ding SW. 2023. Transgene silencing, RNA interference, and the antiviral defense mechanism directed by small interfering RNAs. Phytopathology 113, 616–625. 36441873 10.1094/PHYTO-10-22-0358-IA

[CIT0019] Ershova N , Sheshukova E, Kamarova K, Arifulin E, Tashlitsky V, Serebryakova M, Komarova T. 2022. *Nicotiana benthamiana* Kunitz peptidase inhibitor-like protein involved in chloroplast-to-nucleus regulatory pathway in plant–virus interaction. Frontiers in Plant Science 13, 1041867. 36438111 10.3389/fpls.2022.1041867 PMC9685412

[CIT0020] Fernandez-Calvino L , Faulkner C, Walshaw J, Saalbach G, Bayer E, Benitez-Alfonso Y, Maule A. 2011. Arabidopsis plasmodesmal proteome. PLoS One 6, e18880. 21533090 10.1371/journal.pone.0018880 PMC3080382

[CIT0021] Fichman Y , Myers RJ Jr, Grant DG, Mittler R. 2021. Plasmodesmata-localized proteins and ROS orchestrate light-induced rapid systemic signaling in Arabidopsis. Science Signaling 14, eabf0322. 33622982 10.1126/scisignal.abf0322

[CIT0022] Fichman Y , Zandalinas SI, Peck S, Luan S, Mittler R. 2022. HPCA1 is required for systemic reactive oxygen species and calcium cell-to-cell signaling and plant acclimation to stress. The Plant Cell 34, 4453–4471. 35929088 10.1093/plcell/koac241 PMC9724777

[CIT0023] Fuchs M , van Bel AJ, Ehlers K. 2010. Season-associated modifications in symplasmic organization of the cambium in *Populus nigra*. Annals of Botany 105, 375–387. 20045870 10.1093/aob/mcp300 PMC2826250

[CIT0024] Gao C , Liu X, De Storme N, et al. 2020. Directionality of plasmodesmata-mediated transport in arabidopsis leaves supports auxin channeling. Current Biology 30, 1970–1977. 32275878 10.1016/j.cub.2020.03.014

[CIT0025] Gao X , Yuan Y, Liu Z, Liu C, Xin H, Zhang Y, Gai S. 2021. Chilling and gibberellin acids hyperinduce beta-1,3-glucanases to reopen transport corridor and break endodormancy in tree peony (*Paeonia suffruticosa*). Plant Physiology and Biochemistry 167, 771–784. 34530322 10.1016/j.plaphy.2021.09.002

[CIT0026] Gombos S , Miras M, Howe V, et al. 2023. A high-confidence *Physcomitrium patens* plasmodesmata proteome by iterative scoring and validation reveals diversification of cell wall proteins during evolution. New Phytologist 238, 637–653. 36636779 10.1111/nph.18730

[CIT0027] Grison MS , Brocard L, Fouillen L, et al. 2015. Specific membrane lipid composition is important for plasmodesmata function in Arabidopsis. The Plant Cell 27, 1228–1250. 25818623 10.1105/tpc.114.135731 PMC4558693

[CIT0028] Gunning BES. 1976. Introduction to plasmodesmata. In: Gunning BES, Robards AW, eds. Intercellular comminucation in plants: studies on plasmodesmata. Berlin Heidelberg: Springer-Verlag, 1–13.

[CIT0029] Ham BK , Wang X, Toscano-Morales R, Lin J, Lucas WJ. 2023. Plasmodesmal endoplasmic reticulum proteins regulate intercellular trafficking of cucumber mosaic virus in Arabidopsis. Journal of Experimental Botany 74, 4401–4414. 37210666 10.1093/jxb/erad190 PMC10838158

[CIT0030] Han X , Hyun TK, Zhang M, Kumar R, Koh EJ, Kang BH, Lucas WJ, Kim JY. 2014. Auxin–callose-mediated plasmodesmal gating is essential for tropic auxin gradient formation and signaling. Developmental Cell 28, 132–146. 24480642 10.1016/j.devcel.2013.12.008

[CIT0031] Heinlein M. 2015. Plant virus replication and movement. Virology 479–480, 657–671. 10.1016/j.virol.2015.01.025 25746797

[CIT0032] Huang C , Sede AR, Elvira-Gonzalez L, Yan Y, Rodriguez ME, Mutterer J, Boutant E, Shan L, Heinlein M. 2023. dsRNA-induced immunity targets plasmodesmata and is suppressed by viral movement proteins. The Plant Cell 35, 3845–3869. 37378592 10.1093/plcell/koad176 PMC10533371

[CIT0033] Ishikawa K , Tamura K, Fukao Y, Shimada T. 2020. Structural and functional relationships between plasmodesmata and plant endoplasmic reticulum–plasma membrane contact sites consisting of three synaptotagmins. New Phytologist 226, 798–808. 31869440 10.1111/nph.16391

[CIT0034] Jin T , Wu H, Deng Z, Cai T, Li J, Liu Z, Waterhouse PM, White RG, Liang D. 2022. Control of root-to-shoot long-distance flow by a key ROS-regulating factor in Arabidopsis. Plant, Cell & Environment 45, 2476–2491. 10.1111/pce.14375 35689480

[CIT0035] Johnston MG , Breakspear A, Samwald S, Zhang D, Papp D, Faulkner C, de Keijzer J. 2023. Comparative phyloproteomics identifies conserved plasmodesmal proteins. Journal of Experimental Botany 74, 1821–1835. 36639877 10.1093/jxb/erad022 PMC10049917

[CIT0036] Kirk P , Amsbury S, German L, Gaudioso-Pedraza R, Benitez-Alfonso Y. 2022. A comparative meta-proteomic pipeline for the identification of plasmodesmata proteins and regulatory conditions in diverse plant species. BMC Biology 20, 128. 35655273 10.1186/s12915-022-01331-1 PMC9164936

[CIT0037] Korner CJ , Klauser D, Niehl A, Dominguez-Ferreras A, Chinchilla D, Boller T, Heinlein M, Hann DR. 2013. The immunity regulator BAK1 contributes to resistance against diverse RNA viruses. Molecular Plant-Microbe Interactions 26, 1271–1280. 23902263 10.1094/MPMI-06-13-0179-R

[CIT0038] Lang J , Colcombet J. 2020. Sustained incompatibility between MAPK signaling and pathogen effectors. International Journal of Molecular Sciences 21, 7954. 33114762 10.3390/ijms21217954 PMC7672596

[CIT0039] Lee JY , Wang X, Cui W, et al. 2011. A plasmodesmata-localized protein mediates crosstalk between cell-to-cell communication and innate immunity in Arabidopsis. The Plant Cell 23, 3353–3373. 21934146 10.1105/tpc.111.087742 PMC3203451

[CIT0040] Leijon F , Melzer M, Zhou Q, Srivastava V, Bulone V. 2018. Proteomic analysis of plasmodesmata from Populus cell suspension cultures in relation with callose biosynthesis. Frontiers in Plant Science 9, 1681. 30510561 10.3389/fpls.2018.01681 PMC6252348

[CIT0041] Li J , Yang J, Gao Y, Zhang Z, Gao C, Chen S, Liesche J. 2023. Parallel auxin transport via PINs and plasmodesmata during the Arabidopsis leaf hyponasty response. Plant Cell Reports 43, 4. 38117314 10.1007/s00299-023-03119-1 PMC10733227

[CIT0042] Li Z , Variz H, Chen Y, Liu SL, Aung K. 2021. Plasmodesmata-dependent intercellular movement of bacterial effectors. Frontiers in Plant Science 12, 640277. 33959138 10.3389/fpls.2021.640277 PMC8095247

[CIT0043] Liang D , White RG, Waterhouse PM. 2012. Gene silencing in Arabidopsis spreads from the root to the shoot, through a gating barrier, by template-dependent, nonvascular, cell-to-cell movement. Plant Physiology 159, 984–1000. 22582134 10.1104/pp.112.197129 PMC3387722

[CIT0044] Liang D , White RG, Waterhouse PM. 2014. Mobile gene silencing in Arabidopsis is regulated by hydrogen peroxide. PeerJ 2, e701. 25551023 10.7717/peerj.701 PMC4277490

[CIT0045] Linh NM , Scarpella E. 2022. Leaf vein patterning is regulated by the aperture of plasmodesmata intercellular channels. PLoS Biology 20, e3001781. 36166438 10.1371/journal.pbio.3001781 PMC9514613

[CIT0046] Liu J , Liu Y, Wang S, Cui Y, Yan D. 2022. Heat stress reduces root meristem size via induction of plasmodesmal callose accumulation inhibiting phloem unloading in Arabidopsis. International Journal of Molecular Sciences 23, 2063. 35216183 10.3390/ijms23042063 PMC8879574

[CIT0047] Luna GR , Li J, Wang X, Liao L, Lee JY. 2023. Targeting of plasmodesmal proteins requires unconventional signals. The Plant Cell 35, 3035–3052. 37225403 10.1093/plcell/koad152 PMC10396362

[CIT0048] Luo KR , Huang NC, Chang YH, Jan YW, Yu TS. 2024. Arabidopsis cyclophilins direct intracellular transport of mobile mRNA via organelle hitchhiking. Nature Plants 10, 161–171. 38177664 10.1038/s41477-023-01597-5

[CIT0049] Lv M , Dai Y, Xie L, Guo J, Liao Z, Shang W, Zhao X, Hong J, Zhang HM. 2024. Volume electron microscopy reconstruction uncovers a physical barrier that limits virus to phloem. New Phytologist 241, 343–362. 37858933 10.1111/nph.19319

[CIT0050] Mehra P , Pandey BK, Melebari D, et al. 2022. Hydraulic flux-responsive hormone redistribution determines root branching. Science 378, 762–768. 36395221 10.1126/science.add3771

[CIT0051] Mellor NL , Voss U, Janes G, Bennett MJ, Wells DM, Band LR. 2020. Auxin fluxes through plasmodesmata modify root-tip auxin distribution. Development 147, dev181669. 32229613 10.1242/dev.181669 PMC7132777

[CIT0052] Nicolas WJ , Grison MS, Trepout S, Gaston A, Fouche M, Cordelieres FP, Oparka K, Tilsner J, Brocard L, Bayer EM. 2017. Architecture and permeability of post-cytokinesis plasmodesmata lacking cytoplasmic sleeves. Nature Plants 3, 17082. 28604682 10.1038/nplants.2017.82

[CIT0053] Niehl A , Heinlein M. 2019. Perception of double-stranded RNA in plant antiviral immunity. Molecular Plant Pathology 20, 1203–1210. 30942534 10.1111/mpp.12798 PMC6715784

[CIT0054] Niehl A , Wyrsch I, Boller T, Heinlein M. 2016. Double-stranded RNAs induce a pattern-triggered immune signaling pathway in plants. New Phytologist 211, 1008–1019. 27030513 10.1111/nph.13944

[CIT0055] Ohtsu M , Jennings J, Johnston M, Breakspear A, Liu X, Stark K, Morris RJ, de Keijzer J, Faulkner C. 2024. Assaying effector cell-to-cell mobility in plant tissues identifies hypermobility and indirect manipulation of plasmodesmata. Molecular Plant-Microbe Interactions 37, 84–92. 37942798 10.1094/MPMI-05-23-0052-TA

[CIT0056] Olatunji D , Clark NM, Kelley DR. 2023. The class VIII myosin ATM1 is required for root apical meristem function. Development 150, dev201762. 37306290 10.1242/dev.201762 PMC10357013

[CIT0057] Pan W , Li J, Du Y, et al. 2023. Epigenetic silencing of callose synthase by VIL1 promotes bud-growth transition in lily bulbs. Nature Plants 9, 1451–1467. 37563458 10.1038/s41477-023-01492-z

[CIT0058] Parra-Aguilar TJ , Sarmiento-Lopez LG, Santana O, Olivares JE, Pascual-Morales E, Jimenez-Jimenez S, Quero-Hostos A, Palacios-Martinez J, Chavez-Martinez AI, Cardenas L. 2023. TETRASPANIN 8-1 from *Phaseolus vulgaris* plays a key role during mutualistic interactions. Frontiers in Plant Science 14, 1152493. 37465390 10.3389/fpls.2023.1152493 PMC10352089

[CIT0059] Petit JD , Immel F, Lins L, Bayer EM. 2019. Lipids or proteins: who is leading the dance at membrane contact sites?. Frontiers in Plant Science 10, 198. 30846999 10.3389/fpls.2019.00198 PMC6393330

[CIT0060] Raffaele S , Bayer E, Lafarge D, et al. 2009. Remorin, a solanaceae protein resident in membrane rafts and plasmodesmata, impairs potato virus X movement. The Plant Cell 21, 1541–1555. 19470590 10.1105/tpc.108.064279 PMC2700541

[CIT0061] Rahman MS , Madina MH, Plourde MB, Dos Santos KCG, Huang X, Zhang Y, Laliberte JF, Germain H. 2021. The fungal effector Mlp37347 alters plasmodesmata fluxes and enhances susceptibility to pathogen. Microorganisms 9, 1232. 34204123 10.3390/microorganisms9061232 PMC8228402

[CIT0062] Reagan BC , Burch-Smith TM. 2020. Viruses reveal the secrets of plasmodesmal cell biology. Molecular Plant-Microbe Interactions 33, 26–39. 31715107 10.1094/MPMI-07-19-0212-FI

[CIT0063] Renzaglia K , Duran E, Sagwan-Barkdoll L, Henry J. 2024. Callose in leptoid cell walls of the moss Polytrichum and the evolution of callose synthase across bryophytes. Frontiers in Plant Science 15, 1357324. 38384754 10.3389/fpls.2024.1357324 PMC10879339

[CIT0064] Rinne PL , van der Schoot C. 1998. Symplasmic fields in the tunica of the shoot apical meristem coordinate morphogenetic events. Development 125, 1477–1485. 9502728 10.1242/dev.125.8.1477

[CIT0065] Rinne PLH , Paul LK, van der Schoot C. 2018. Decoupling photo- and thermoperiod by projected climate change perturbs bud development, dormancy establishment and vernalization in the model tree Populus. BMC Plant Biology 18, 220. 30290771 10.1186/s12870-018-1432-0 PMC6173867

[CIT0066] Sager R , Wang X, Hill K, Yoo BC, Caplan J, Nedo A, Tran T, Bennett MJ, Lee JY. 2020. Auxin-dependent control of a plasmodesmal regulator creates a negative feedback loop modulating lateral root emergence. Nature Communications 11, 364. 10.1038/s41467-019-14226-7 PMC6969147 31953391

[CIT0067] Schapire AL , Voigt B, Jasik J, et al. 2008. Arabidopsis synaptotagmin 1 is required for the maintenance of plasma membrane integrity and cell viability. The Plant Cell 20, 3374–3388. 19088329 10.1105/tpc.108.063859 PMC2630439

[CIT0068] Schreier TB , Muller KH, Eicke S, Faulkner C, Zeeman SC, Hibberd JM. 2024. Plasmodesmal connectivity in C_4_*Gynandropsis gynandra* is induced by light and dependent on photosynthesis. New Phytologist 241, 298–313. 37882365 10.1111/nph.19343 PMC10952754

[CIT0069] Schroder F , Lisso J, Mussig C. 2012. Expression pattern and putative function of EXL1 and homologous genes in Arabidopsis. Plant Signaling and Behavior 7, 22–27. 22301961 10.4161/psb.7.1.18369 PMC3357360

[CIT0070] Simon-Plas F , Perraki A, Bayer E, Gerbeau-Pissot P, Mongrand S. 2011. An update on plant membrane rafts. Current Opinion in Plant Biology 14, 642–649. 21903451 10.1016/j.pbi.2011.08.003

[CIT0071] Slupianek A , Myskow E, Kasprowicz-Maluski A, Dolzblasz A, Zytkowiak R, Turzanska M, Sokolowska K. 2023. Seasonal dynamics of cell-to-cell transport in angiosperm wood. Journal of Experimental Botany 75, 1331–1346. 10.1093/jxb/erad469 PMC10901208 37996075

[CIT0072] Talbi, N, Blekemolen MC, Janevska S, Zendler DP, Van Tilbeurgh H, Fudal I, Takken FLW. 2023. Facilitation of symplastic effector protein mobility by paired effectors is conserved in different classes of fungal pathogens. Molecular Plant-Microbe Interactions 37, 304–314. 10.1094/MPMI-07-23-0103-FI 37782126

[CIT0073] Tee EE , Johnston MG, Papp D, Faulkner C. 2023. A PDLP–NHL3 complex integrates plasmodesmal immune signaling cascades. Proceedings of the National Academy of Sciences, USA 120, e2216397120. 10.1073/pnas.2216397120 PMC10151459 37068237

[CIT0074] Thomas CL , Bayer EM, Ritzenthaler C, Fernandez-Calvino L, Maule AJ. 2008. Specific targeting of a plasmodesmal protein affecting cell-to-cell communication. PLoS Biology 6, e7. 18215111 10.1371/journal.pbio.0060007 PMC2211546

[CIT0075] Tilney LG , Cooke TJ, Connelly PS, Tilney MS. 1991. The structure of plasmodesmata as revealed by plasmolysis, detergent extraction, and protease digestion. Journal of Cell Biology 112, 739–747. 1993740 10.1083/jcb.112.4.739 PMC2288846

[CIT0076] Tilsner J , Amari K, Torrance L. 2011. Plasmodesmata viewed as specialised membrane adhesion sites. Protoplasma 248, 39–60. 20938697 10.1007/s00709-010-0217-6

[CIT0077] Tilsner J , Kriechbaumer V. 2022. Reticulons 3 and 6 interact with viral movement proteins. Molecular Plant Pathology 23, 1807–1814. 35987858 10.1111/mpp.13261 PMC9644274

[CIT0078] Tomczynska I , Stumpe M, Doan TG, Mauch F. 2020. A Phytophthora effector protein promotes symplastic cell-to-cell trafficking by physical interaction with plasmodesmata-localised callose synthases. New Phytologist 227, 1467–1478. 32396661 10.1111/nph.16653

[CIT0079] Toyota M , Spencer D, Sawai-Toyota S, Jiaqi W, Zhang T, Koo AJ, Howe GA, Gilroy S. 2018. Glutamate triggers long-distance, calcium-based plant defense signaling. Science 361, 1112–1115. 30213912 10.1126/science.aat7744

[CIT0080] Tylewicz S , Petterle A, Marttila S, et al. 2018. Photoperiodic control of seasonal growth is mediated by ABA acting on cell–cell communication. Science 360, 212–215. 29519919 10.1126/science.aan8576

[CIT0081] Vaattovaara A , Brandt B, Rajaraman S, et al. 2019. Mechanistic insights into the evolution of DUF26-containing proteins in land plants. Communications Biology 2, 56. 30775457 10.1038/s42003-019-0306-9 PMC6368629

[CIT0082] Vu MH , Hyun TK, Bahk S, Jo Y, Kumar R, Thiruppathi D, Iswanto ABB, Chung WS, Shelake RM, Kim JY. 2023. ROS-mediated plasmodesmal regulation requires a network of an Arabidopsis receptor-like kinase, calmodulin-like proteins, and callose synthases. Frontiers in Plant Science 13, 1107224. 36743578 10.3389/fpls.2022.1107224 PMC9893415

[CIT0083] Wang P , Hawkins TJ, Richardson C, Cummins I, Deeks MJ, Sparkes I, Hawes C, Hussey PJ. 2014. The plant cytoskeleton, NET3C, and VAP27 mediate the link between the plasma membrane and endoplasmic reticulum. Current Biology 24, 1397–1405. 24909329 10.1016/j.cub.2014.05.003

[CIT0084] Wang P , Khoshravesh R, Karki S, Tapia R, Balahadia CP, Bandyopadhyay A, Quick WP, Furbank R, Sage TL, Langdale JA. 2017. Re-creation of a key step in the evolutionary switch from C_3_ to C_4_ leaf anatomy. Current Biology 27, 3278–3287. 29056456 10.1016/j.cub.2017.09.040 PMC5678070

[CIT0085] Wang Y , Perez-Sancho J, Platre MP, et al. 2023. Plasmodesmata mediate cell-to-cell transport of brassinosteroid hormones. Nature Chemical Biology 19, 1331–1341. 37365405 10.1038/s41589-023-01346-x PMC10729306

[CIT0086] Xu B , Cheval C, Laohavisit A, Hocking B, Chiasson D, Olsson TSG, Shirasu K, Faulkner C, Gilliham M. 2017. A calmodulin-like protein regulates plasmodesmal closure during bacterial immune responses. New Phytologist 215, 77–84. 28513846 10.1111/nph.14599 PMC5488192

[CIT0087] Yuan C , Lazarowitz SG, Citovsky V. 2016. Identification of a functional plasmodesmal localization signal in a plant viral cell-to-cell-movement protein. MBio 7, e02052-15. 10.1128/mBio.02052-15 PMC4725018 26787834

[CIT0088] Yuan C , Lazarowitz SG, Citovsky V. 2018. The plasmodesmal localization signal of TMV MP is recognized by plant synaptotagmin SYTA. MBio 9, e01314-18. 29991585 10.1128/mBio.01314-18 PMC6050956

[CIT0089] Zavaliev R , Dong X, Epel BL. 2016. Glycosylphosphatidylinositol (GPI) modification serves as a primary plasmodesmal sorting signal. Plant Physiology 172, 1061–1073. 27559035 10.1104/pp.16.01026 PMC5047108

[CIT0090] Zhang Y , Berman A, Shani E. 2023. Plant hormone transport and localization: signaling molecules on the move. Annual Review of Plant Biology 74, 453–479. 10.1146/annurev-arplant-070722-015329 36889002

